# Ankle Brachial Index in Different Types of Popliteal Artery Entrapment Syndrome: A Systematic Review of Case Reports

**DOI:** 10.3390/jcm8122071

**Published:** 2019-11-26

**Authors:** Felice Sirico, Stefano Palermi, Francesco Gambardella, Eduardo Capuano, Umberto Ferrari, Veronica Baioccato, Clotilde Castaldo, Franca Di Meglio, Daria Nurzynska

**Affiliations:** University of Naples Federico II, Department of Public Health, Human Anatomy and Sports Medicine Division, 80131 Naples, Italy

**Keywords:** popliteal artery, ankle brachial index, peripheral artery disease, variation, skeletal muscle

## Abstract

Similar to other peripheral artery diseases, vessel narrowing in popliteal artery entrapment syndrome (PAES) reduces the ankle brachial index (ABI). Since the PAES is related to several anatomical or functional variations, we sought to determine if the ABI was correlated with the type of syndrome. Through a systematic review of literature, we identified case reports and series in which the diagnosis of PAES was accompanied by ABI measurement. Twenty-seven studies included in the qualitative synthesis described 87 limbs. The most common types of the syndrome were those caused by an abnormal medial head of the gastrocnemius (type II, *n* = 35, 40.23%) and aberrant course of the popliteal artery (type I, *n* = 20, 22.99%). The variation of plantaris muscle (*n* = 7, 8.05%) is currently not included in the classification system. The median value of ABI was 0.87 (interquartile range (IQR) = 0.6–1.0). There were no differences among types of syndrome (F = 0.13, *p* = 0.72). In conclusion, despite clinical recommendations, the ABI remains underused in PAES diagnosis. No correlation was detected between the index score and type of syndrome. The cases of PAES involving structures other than the gastrocnemius or popliteus muscle suggest the need to revisit the current clinical classification system.

## 1. Introduction

Musculoskeletal injury is the most common cause of pain and dysfunction among sports participants, particularly in young males [1]. Vascular disease, however, may present with similar symptoms, and should always be excluded by taking a complete history and performing a physical examination [2]. One of the possible causes of claudication in healthy young adults without atherosclerotic risk factors is popliteal artery entrapment syndrome (PAES), which causes acute lower limb ischemia. PAES is a congenital pathology caused by anatomical abnormalities in the popliteal fossa. In particular, the popliteal artery can be compressed by adjacent musculotendinous structures, commonly the gastrocnemius or popliteal muscles. The entrapment is frequently diagnosed in athletes, likely due to the hypertrophy of the gastrocnemius muscle and higher demands on the lower extremity circulation in activities such as basketball, football, rugby, and martial arts [3]. PAES is a progressive and debilitating disease in which repetitive mechanical compression of the artery can cause severe destruction of the artery wall. Subsequent intraluminal stenosis, poststenotic dilatation, and aneurysm formation can occur, which may lead to thrombus formation, embolization, and limb ischemia. Accordingly, correct and early diagnosis of this syndrome is important in preventing deterioration of the artery wall [4].

The ankle brachial index (ABI) is the first-line test for diagnosing peripheral artery disease [5,6,7]. The ABI is a non-invasive and inexpensive method that has high sensitivity and specificity compared to the current gold standard of invasive angiography. The ABI is obtained by measuring the systolic blood pressure at the arm (brachial arteries) and ankle (dorsalis pedis or posterior tibial arteries) in the supine position using a Doppler probe or an oscillometric device [8]. The pressure in the lower limbs is normally slightly higher than the pressure in the upper limbs. Therefore, the ratio between these two pressure measurements is considered normal between 1 and 1.4 [9]. In peripheral artery disease, the ABI score is useful both to confirm the diagnosis and to grade disease severity. Clinically, an ABI ≤ 0.9 indicates peripheral artery disease, which can then be graded as mild to moderate if it ranges between 0.9 and 0.4, or severe if it is lower than 0.4 [10]. However, a score greater than 1.4 is also considered pathological, as it suggests calcifying noncompressible arterial disease, and is associated with higher cardiovascular mortality [11]. Importantly, Fowkes et al. [12] introduced a model for improved cardiovascular risk prediction based on the ABI and Framingham score. For PAES, the symptoms and physical examination findings should also be confirmed by ABI measurements [13]. Additionally, a stress test consisting of the ABI measurement in plantar flexion with the knee fully extended is often positive in PAES. Determination of ABI alone, with and without provocation, can be considered sufficient as postoperative examination in patients in whom ABI was 0.9 or lower to confirm decompression of popliteal artery [14].

Despite the increasing recognition of popliteal artery entrapment as the cause of lower limb ischemia, differentiation between several types of PAES remains a preoperative diagnostic challenge [15] due to the quantity and complexity of the triggering anatomical variations and limitations of diagnostic imaging techniques. A widely accepted and currently used classification of PAES was proposed by Love and Whelan [16] and modified by Rich et al. [17]. According to this system, type I is associated with an aberrant medial arterial course around the normal medial head of the gastrocnemius muscle. In type II, an abnormal medial head of the gastrocnemius inserts laterally on the distal femur and displaces the popliteal artery medially. Type III is depicted by an aberrant accessory slip from the medial head of the gastrocnemius muscle that wraps around the popliteal artery. In type IV, the popliteal artery is entrapped by the popliteus muscle. In type V, the popliteal vein is also involved. Type VI is considered functional and is recognized in the presence of a normally positioned popliteal artery that is entrapped by a normally positioned but hypertrophied gastrocnemius muscle.

As mentioned above, ABI is the simplest test, allowing the initial evaluation for peripheral artery disease. It has been suggested that the severity of lower limb arterial disease can be graded and the risk for cardiovascular events (such as cardiac ischemia or stroke) can be predicted based on the estimated ABI [10,12]. While the popliteal artery entrapment is a heterogeneous syndrome not associated with the systemic disease and cardiovascular risk factors, it can still be a cause of serious disability due to local circulatory insufficiency and arterial wall damage. Taking into account the variations in its anatomical and clinical presentation, we investigated the relation between ABI score and PAES type, aiming at providing two diagnostic cues: first, whether a particular type of PAES presents with lower ABI, and hence higher severity, at diagnosis than the others; and second, whether the ABI score can aid in predicting the type of PAES.

To this end, we examined the relationship between the anatomical variation causing PAES and the ABI score. Through a systematic review of case reports and case series, our study also sheds new light on the anatomical abnormalities causing PAES, indicating a need to revisit the current classification system.

## 2. Experimental Section

The following electronic databases were searched from inception to March 2019: PubMed, Web of Science, Scopus, Medline, and CINAHL (Cumulative Index to Nursing and Allied Health Literature). Based on the syntax rules of each database, the following terms were combined to retrieve all studies relevant to the main topic of analysis: popliteal, artery, entrapment, syndrome, anatomy, PAES, ABI, and ankle brachial index. The results were collected using reference manager software. After the removal of duplicates, three authors screened the articles for relevance based on the title and abstract. All inconsistencies were discussed with another author. The same two authors independently analyzed the full text of the remaining articles to determine their eligibility. Additionally, the reference lists of relevant articles were scanned to identify other published and non-indexed sources. The following inclusion criteria were applied: case report or case series, definitive diagnosis of PAES at the end of the diagnostic or therapeutic phase, measurement of the ABI score of every included patient before treatment, and full text written in English. Observational studies, cohort studies, and narrative and systematic reviews were excluded.

The extracted data included the name of the first author, year of publication, country of origin, number of reported cases, number of limbs, age and sex of the patients, type of PAES, ABI score before the intervention, and technique used for the ABI measurement. The type of diagnosed PAES was reported according to the previously cited classification and was extracted from the included studies directly, or if explicit information about PAES type was missing, was based on the interpretation of the anatomical description reported within the study. Because a specific tool dedicated to the assessment and critical appraisal of case reports and case series in anatomical sciences is not available, the studies included in our analysis were assessed using the Joanna Briggs Institute (JBI) checklist for case reports [18].

All analyses were performed using Stata software v.12 (StataCorp LLC, College Station (TX), USA)). Sex distribution and type of PAES are reported as frequencies and percentages. Age is reported as the mean with standard deviation (SD). The distribution of the ABI scores was analyzed using the Shapiro–Wilk test and is reported as the median with interquartile range (IQR). For the comparison of the ABI scores among different types of PAES, data were transformed to meet the assumption of normality and analyzed by parametric test. Squared ABI score was the mathematical transformation able to produce a near-normal distribution. Therefore, squared ABI scores were used to perform one-way ANOVA, with ABI scores as a dependent variable and type of PAES as an independent variable. Pairwise comparisons were conducted only if significant difference was evident. A value of *p* < 0.05 was considered statistically significant.

## 3. Results

**Figure 1:**
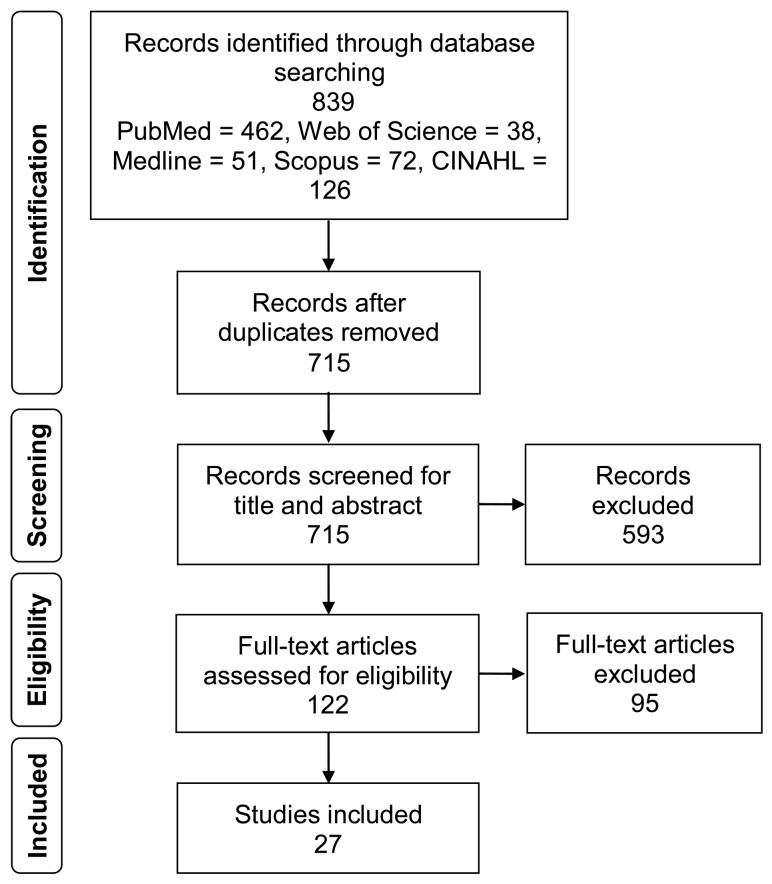
Preferred reporting items for systematic reviews and meta-analyses (PRISMA) flowchart of the study selection process.

An adapted PRISMA flowchart (Figure 1) summarizes the results of the identification, screening, and eligibility evaluation of the studies. The preferred reporting items for systematic reviews and meta-analyses (PRISMA) checklist [19] is available online as Supplementary Table S1.

Through a database search, 839 articles were identified. After removal of duplicates and title and abstract screening, 122 articles had their full text retrieved and were assessed for eligibility based on the inclusion criteria. Of these, 95 were deemed unsuitable for one of the following reasons: missing pre-treatment ABI score (83 reports), the article was not a case report (11 reports), or not in English (1 report). As a result, 27 reports were included in the qualitative analysis. The characteristics of the included case studies are summarized in Table 1.

The studies included in our analysis were assessed using the checklist developed by Moola et al. [18], and the results are summarized in Table 2. Only three (10.7%) studies clearly reported all the elements of the research evidence. Patients’ medical, family, and psychosocial histories, including relevant genetic information, were not reported in 33.3% of the studies and were unclear in 11.1% of the reports. All included studies described the clinical condition of the patient at the time of presentation and used diagnostic tests, providing relevant photographs or illustrations. Treatment or intervention procedures were presented in detail in 96.3% of the studies, while the clinical symptoms during post-intervention were reported in 74.1% of the studies. Adverse events were specifically assessed in just over half (53.6%) of the reports. Finally, the key points were summarized as a takeaway lesson in a minority (44.5%) of cases.

Overall, 62 patients and 87 limbs with PAES were described in the included case reports. The majority of patients were male (*n* = 50, 80.6%). The mean age was 32.63 years (SD 18.76). Entrapment was present only in the right limb in 24 patients (38.7%) and only in the left limb in 13 patients (20.97%). Both lower limbs were involved in 25 cases (40.32%), and 8% of those patients had different types of PAES in the right and left limbs. The results, as shown in Table 3, indicated that the most common types of PAES were those caused by an abnormal medial head of the gastrocnemius that displaced the popliteal artery (type II, *n* = 35, 40.22%) and an aberrant course of the popliteal artery around the normal medial head of the gastrocnemius muscle (type I, *n* = 20, 22.98%). There were no reports of popliteal vein entrapment (type V). Among cases that could not be classified (*n* = 11, 12.64%) according to the current classification system, seven patients had an aberrant plantaris muscle.

The method used for the ABI evaluation was not specified in 67.86% of the publications. One study [21] reported the ABI both at rest and during stress tests; we considered the value at rest for statistical analysis. In one case, only the ABI measured during a stress test in plantar flexion was reported [25], while in six studies, the measurements were taken at rest [33,35,39,40,41,46].

The median value of all ABI scores was 0.87 (IQR = 0.6–1.0). In only eight out of 87 limbs with PAES was the ABI score greater than 1, with the highest reported value equal to 1.2. Remarkably, patient had an ABI < 1 in dorsal rather than in plantar flexion. In the other eight limbs, the ABI scores were lower than 0.4, indicating a severe condition. In a single case, an ABI = 0 was reported [29], and that patient suffered acute lower limb ischemia. 

The ABI score distribution was not normal and appeared left-skewed due to the rare recordings of the extremely low scores. When normally transformed ABI scores were used in parametric tests, there were no significant differences in the ABI scores among different types of PAES (F = 0.13, *p* = 0.72). Overall, no association was detected between the ABI and the type of PAES.

## 4. Discussion

The current analysis of a series of case reports did not find any evidence that ABI score was correlated with the type of PAES. Although the ABI was abnormal in most cases, a greater or smaller decrease or increase in the index score at diagnosis was not related to a specific type of PAES. Accordingly, a clear association between the PAES type and disease severity, as expressed by the ABI score at diagnosis, could not be identified. One unanticipated finding was that the current classification system of PAES did not cover all cases, as several of them were caused by an aberrant plantaris muscle.

Although PAES remains a rare cause of vascular disease, its incidence has increased in recent years [47]. Therefore, PAES should be considered in the differential diagnosis of patients with lower limb pain, in addition to other vascular, muscle/fascia, bone/periosteum, and peripheral nerve diseases. Differentiation among several types of PAES is challenging due to the variability and complexity of the involved anatomical structures and limitations of diagnostic imaging techniques. An analysis by Clemens et al. [15] found that patients with functional entrapment and PAES type VI, in whom a musculotendinous slip was not found intraoperatively, were least likely to demonstrate clinical improvement. Consequently, not all types of PAES can be resolved surgically, and early identification of those cases should allow personalization of the approach, possibly reducing the time and cost of patient management.

A prior study [48] noted that type 1 and type 2 PAES occurred most frequently, and our findings based on a series of case reports published between 1990 and 2018 confirm that observation. Regarding other types of this syndrome, our analysis revealed that entrapment caused by the plantaris muscle had a higher prevalence than anomalies typical of PAES types IV, V, or VI. Importantly, in the commonly adopted classification of PAES, anomalies are related to the artery course (type I), gastrocnemius muscle (type II and III), popliteus muscle (type IV), or popliteal vein (type V), while the presence of an aberrant plantaris muscle has not been considered so far. The relatively high prevalence of this “unclassified” type of PAES suggests the need to revisit the current clinical classification system.

The role of ABI measurement in the diagnosis of peripheral arterial disease is well established. The ABI is a very sensible diagnostic method, which when used properly, reduces the time to treatment initiation [4]. According to the American Heart Association and American College of Cardiology guidelines [7], the measurement of ABI at rest is recommended to establish a diagnosis in patients with atherosclerosis risk factors and with (class I recommendation) or without (class II recommendation) history or physical examination findings suggestive of peripheral arterial disease. The Society for Vascular Surgery recommends the ABI measurement at rest and after exercise in patients with claudication [6]. In a prospective cohort study, McDermott et al. [49] found that lower ABI values were associated with a greater incidence of lower limb disability, as expressed by walking speed and distance. However, most reports identified in the literature search failed to include the ABI score and had to be excluded from our analysis. This indicates that the clinical utility of this index is often underestimated.

Rare ABI reporting in published PAES cases certainly limited the sample size in our study. With a small sample size, caution must be applied, as the findings may not be transferable to the wider population. Another limitation of the present analysis is related to the design of the included studies, as case reports and case series are intrinsically associated with publication bias. Admittedly, scientific robustness of the resulting data is far from the methodological rigor of a systematic review and meta-analysis of controlled clinical studies. However, several researchers [50,51,52] demonstrated that a methodological synthesis of case reports and case series could provide a useful addition to evidence-based medicine, particularly in the field of rare and heterogeneous conditions. Indeed, a systematic review of case reports or case series is the only available method for assessing the relationship between the PAES type and ABI score. The systematic search of the available literature and a rigorous screening process following PRISMA principles limited some sources of bias in data collection and interpretation. In the authors’ opinion, the scientific approach adopted in the present analysis represents the only valuable methodology to address our hypothesis based on available studies of this rare syndrome.

In patients with peripheral artery disease, the ABI score can be influenced by arterial wall stiffness. While PAES is typically diagnosed in physically active men aged 20 to 40 years without peripheral artery disease, our review of case reports and case series points out that it affects also women and can cause limb ischemia at later age. It can be assumed that atherosclerosis may be present in some, especially older, patients with this diagnosis, and even in the absence of atherosclerosis, the wall damage caused by prolonged compression can result in tissue remodeling and arterial stiffness. However, it is not possible to adjust for arterial stiffness on the basis of data available in the reports included in our systematic review. Another limitation can be related to the fact that all cases involving an aberrant plantaris muscle were described in the same report, which likely represents a selection bias. Nevertheless, the results still provide useful information about the etiology of PAES.

In conclusion, the ABI remains underused in PAES, despite clear clinical recommendations for the diagnosis of peripheral artery disease. Based on available data, there is no correlation between the ABI score and the type of PAES; hence, imaging techniques and surgical exploration remain essential for treatment choice and outcome. The cases of PAES involving structures other than the gastrocnemius or popliteus muscle suggest the need to revisit the current clinical classification system.

## Figures and Tables

**Table 1:** Summary of the characteristics of the studies included in the analysis.

Study	Country	*N*	Sex	Age, Mean (SD)	Side	Classification	ABI, Mean (SD)
Ando et al. [20]	Japan	1	F	65	L	N/C	0.7
Bou and Day [21]	The United States	1	F	18	L	N/C	1.19
Collins et al. [22]	The United States	11	9 M, 2 F	28.5 (10.2)	7 R+L, 3 R, 1 L	1 type I, 6 type II, 5 type III, 6 type IV	0.92 (0.15)
Filis et al. [23]	Greece	1	M	27	R	I	0.62
Gemayel et al. [24]	Switzerland	1	M	27	R	II	0.72
Gocen et al. [25]	Turkey	1	M	36	1 R+L	2 type I	0.89 (0.03)
Guthrie et al. [26]	The United Kingdom	1	M	19	L	I	0.3
Imtiaz [27]	Pakistan	1	M	32	R	N/S	0.5
Iwaki et al. [28]	Japan	1	M	59	1 R+L	2 type I	0.76 (0.21)
Iwasaki et al. [29]	Japan	1	M	73	R	III	0
Jikuya et al. [30]	Japan	1	M	22	1 R+L	2 type II	0.61 (0.23)
Jo and Bae [31]	South Korea	1	M	30	R	II	0.48
Kamphuis et al. [32]	Netherlands	1	M	17	1 R+L	2 type I	0.9 (0.42)
Kukreja et al. [33]	The United States	1	F	18	1 R+L	2 type VI	1 (0)
Kwon et al. [34]	South Korea	6	5 M, 1 F	32.2 (13.0)	1 R+L, 3 R, 2 L	N/C	0.62 (0.26)
Lejay et al. [35]	France	18	17 M, 1 F	34.8 (9.72)	7 R+L, 5 R, 6 L	4 type I, 17 type II, 2 type III, 2 type VI	0.83 (0.25)
Mailis et al. [36]	The United States	1	M	50	R	N/C	1
McAree et al. [37]	Northern Ireland	1	M	33	1 R+L	2 type II	0.59 (0.06)
McGuinness et al. [38]	The United States	1	F	19	1 R+L	2 type II	0.92 (0.35)
Öztoprak et al. [39]	Turkey	1	F	22	R	III	0.7
Passias et al. [40]	The United States	1	M	19	R	I	1.02
Politano et al. [41]	The United States	1	F	15	R	I	0.76
Ring et al. [42]	The United States	2	N/S	40 (22.6)	1 R+L, 1 R	3 type II	0.77 (0.13)
Sieunarine et al. [43]	Australia	3	3 M	42 (23.3)	1 L, 2 R	3 type I	0.54 (0.07)
Sirisena et al. [44]	Singapore	1	M	25	R	II	0.87
Sugimoto et al. [45]	Japan	1	M	73	1 R+L	2 type III	0.38 (0.01)
Symeonidis et al. [46]	Australia	1	M	19	1 R+L	2 type I	1 (0)

Note: F, female; L, left; M, male; N/C, not included in the current classification system; N/S, not specified; R, right.

**Table 2:** Critical evaluation of case reports and case series included in the analysis.

Study	Demographic Characteristics	Clinical History	Clinical Condition on Presentation	Diagnostic Tests	Treatment	Post Intervention Conditions	Adverse Events	Takeaway Lessons
Ando et al. [20]	Yes	Yes	Yes	Yes	Yes	Yes	Yes	No
Bou and Day [21]	Yes	No	Yes	Yes	Yes	Yes	Unclear	Yes
Collins et al. [22]	Yes	Unclear	Yes	Yes	Yes	Unclear	Yes	Yes
Filis et al. [23]	Yes	Yes	Yes	Yes	Yes	Yes	No	Yes
Gemayel et al. [24]	Yes	No	Yes	Yes	Yes	Yes	Yes	No
Gocen et al. [25]	Yes	Yes	Yes	Yes	Yes	Yes	Yes	Yes
Guthrie et al. [26]	Yes	No	Yes	Yes	Yes	Yes	No	Yes
Imitiaz [27]	Yes	Yes	Yes	Yes	Yes	Yes	Unclear	No
Iwaki et al. [28]	Yes	Yes	Yes	Yes	Yes	Yes	No	Yes
Iwasaki et al. [29]	Yes	No	Yes	Yes	Yes	Yes	Yes	Yes
Jikuya et al. [30]	Yes	Yes	Yes	Yes	Yes	Yes	Yes	Yes
Jo and Bae [31]	Yes	Yes	Yes	Yes	Yes	Yes	No	No
Kamphuis et al. [32]	Yes	No	Yes	Yes	Yes	Yes	Unclear	No
Kukreja et al. [33]	Yes	Yes	Yes	Yes	Yes	Unclear	No	No
Kwon et al. [34]	Yes	Yes	Yes	Yes	Yes	No	Yes	Yes
Lejay et al. [35]	Yes	Yes	Yes	Yes	Yes	Yes	No	Yes
Mailis et al. [36]	Yes	Yes	Yes	Yes	No	Unclear	No	No
McAree et al. [37]	Yes	Yes	Yes	Yes	Yes	Yes	No	Yes
McGuinness et al. [38]	Yes	Unclear	Yes	Yes	Yes	No	Unclear	No
Öztoprak et al. [39]	Yes	No	Yes	Yes	Yes	Yes	Unclear	No
Passias et al. [40]	Yes	No	Yes	Yes	Yes	No	Yes	No
Politano et al. [41]	Yes	Yes	Yes	Yes	Yes	Yes	Yes	No
Ring et al. [42]	No	No	Yes	Yes	Yes	No	No	No
Sieunarine et al. [43]	Yes	Unclear	Yes	Yes	Yes	Yes	No	No
Sirisena et al. [44]	Yes	Yes	Yes	Yes	Yes	Yes	Yes	Yes
Sugimoto et al. [45]	Yes	Yes	Yes	Yes	Yes	Yes	Yes	No
Symeonidis et al. [46]	Yes	No	Yes	Yes	Yes	Yes	Yes	No

**Table 3:** Frequencies and median ankle brachial index (ABI) scores in different types of popliteal artery entrapment syndrome (PAES).

PAES	*N*	%	Pre-Treatment ABI, Median (Interquartile Range)
Type I	20	22.99	0.78 (0.59–0.96)
Type II	35	40.23	0.90 (0.72–1.00)
Type III	11	12.64	0.70 (0.39–0.97)
Type IV	6	6.90	1.00 (1.00–1.00)
Type V	0	0	-
Type VI	4	4.60	0.95 (0.60–1.00)
Unclassified	11	12.64	0.63 (0.50–0.95)

## Supplementary Materials

The following are available online at https://www.mdpi.com/2077-0383/8/12/2071/s1, Table S1: 2009 checklist.
